# Myocardial Injury after Non-Cardiac Surgery in Patients Who Underwent Open Repair for Abdominal Aortic Aneurysm: A Retrospective Study

**DOI:** 10.3390/jcm13040959

**Published:** 2024-02-07

**Authors:** Myung Il Bae, Tae-Hoon Kim, Hei Jin Yoon, Suk-Won Song, Narhyun Min, Jongyun Lee, Sung Yeon Ham

**Affiliations:** 1 Department of Anesthesiology and Pain Medicine, Yonsei University College of Medicine, Seoul 03722, Republic of Korea; bmi87@yuhs.ac (M.I.B.); phin86@yuhs.ac (H.J.Y.); minna0402@yuhs.ac (N.M.); jongyun27@yuhs.ac (J.L.); 2 Department of Thoracic and Cardiovascular Surgery, Yonsei University College of Medicine, Seoul 03722, Republic of Korea; airtech2@yuhs.ac; 3 Department of Cardiovascular Surgery, Ewha Womans University Aorta and Vascular Hospital, Seoul 07804, Republic of Korea; 4 Anesthesia and Pain Research Institute, Yonsei University College of Medicine, Seoul 03722, Republic of Korea

**Keywords:** myocardial injury after non-cardiac surgery, abdominal aortic aneurysm, abdominal aortic aneurysm open repair, mortality

## Abstract

Background: Myocardial injury after non-cardiac surgery (MINS) has been known to be associated with mortality in various surgical patients; however, its prognostic role in abdominal aortic aneurysm (AAA) open repair remains underexplored. This study aimed to investigate the role of MINS as a predictor of mortality in patients who underwent AAA open repair. Methods: This retrospective study investigated 352 patients who underwent open repair for non-ruptured AAA. The predictors of 30-day and 1-year mortalities were investigated using logistic regression analysis. Results: MINS was diagnosed in 41% of the patients after AAA open repair in this study. MINS was an independent risk factor of 30-day mortality (odds ratio [OR]: 10.440, 95% confidence interval [CI]: 1.278–85.274, *p* = 0.029) and 1-year mortality (OR: 5.189, 95% CI: 1.357–19.844, *p* = 0.016). Kaplan–Meier survival curves demonstrated significantly lower overall survival rates in patients with MINS compared to those without MINS (*p* = 0.003). Conclusion: This study revealed that MINS is a common complication after AAA open repair and is an independent risk factor of 30-day and 1-year mortalities. Patients with MINS have lower overall survival rates than those without MINS.

## 1. Introduction

Abdominal aortic aneurysm (AAA) open repair has been known to be a high-risk vascular surgery with a significant mortality rate [1], and risk stratification among these patients is important. Several risk prediction models for AAA open repair have been developed [2,3]; however, these models primarily focus on preoperative characteristics and do not address the postoperative recovery phase. Recently, postoperative recovery has been reported to be directly related to outcomes in many surgical disciplines [4], and the importance of the recovery phase has been emphasized. Specifically, myocardial injury after non-cardiac surgery (MINS) has been reported to be closely related to mortality in various surgical patients [5,6,7,8].

MINS is defined as troponin elevation by an ischemic etiology within 30 days after non-cardiac surgery [9,10]. It is a broader concept than myocardial infarction, as MINS includes cases in which only troponin levels are elevated without symptoms or electrocardiographic changes [9,10]. Recently, large-scale cohort studies on MINS have been conducted [5,6], and detailed diagnostic criteria for MINS have been introduced [9,10]. These criteria include troponin elevation up to 30 days after surgery as part of MINS while excluding chronic troponin elevation and troponin elevation attributed to non-ischemic causes [9,10]. While the prognostic role of MINS in general vascular surgery has been previously investigated [11], its role in AAA open repair remains underexplored. AAA open repair has different characteristics from other vascular surgeries, including the aortic cross-clamp (ACC) procedure. Therefore, we aimed to investigate the role of MINS as a predictor of mortality in patients who underwent AAA open repair.

## 2. Materials and Methods

This retrospective study was conducted at Gangnam Severance Hospital (Seoul, Republic of Korea). The Institutional Review Board of Yonsei University Gangnam Severance Hospital approved this study on 7 July 2023 (approval number: 3-2023-0187). The study was conducted in accordance with the principles of the Declaration of Helsinki. The need for informed consent was waived because this study was designed as a retrospective study.

### 2.1. Study Population

The study included patients who underwent open repair of AAA at Gangnam Severance Hospital between September 2010 and April 2022. The exclusion criteria were as follows: (1) ruptured AAA, (2) traumatic aortic injury, (3) no troponin T (TnT) results within 30 days after surgery, (4) TnT elevation due to non-ischemic causes (such as atrial fibrillation, sepsis, acute heart failure, pulmonary embolism, and cardioversion), and (5) insufficient medical records.

### 2.2. Data Collection

We collected data on age; sex; body mass index (BMI); smoking history; AAA size; history of percutaneous coronary intervention; preoperative comorbidities, which include hypertension, diabetes mellitus, cerebrovascular accident, coronary artery disease (CAD), peripheral artery occlusive disease, chronic obstructive pulmonary disease, anemia, chronic kidney disease (CKD), and end-stage renal disease (ESRD); preoperative medication, including beta-blockers, calcium channel blockers, renin–angiotensin system inhibitors, statins, and diuretics; and preoperative laboratory results, including high-sensitive TnT, hemoglobin levels, platelet count, white blood cell count, serum C-reactive protein levels, blood urea nitrogen levels, creatinine levels, and estimated glomerular filtration rate. The following intraoperative data were collected: the amount of fluid administered, urine output, estimated amount of bleeding, cell saver volume, operation time, anesthesia time, and ACC time. The following postoperative data were collected: all high-sensitive TnT results for 30 days after surgery, hospital length of stay, intensive care unit (ICU) length of stay, mechanical ventilation time, mortality, and postoperative complications, including cerebrovascular accidents, acute kidney injury (AKI), and myocardial infarction. The definition of AKI follows the guideline of Kidney Disease Improving Global Outcomes [12]. The date of operation, mortality, and the last follow-up were also collected.

### 2.3. Surgical Procedure

At our institution, AAA open repair was performed according to the following protocol. The choice of graft configuration depended on the extent of the aneurysmal disease. Although surgical access to the aorta can be achieved through either a transperitoneal or retroperitoneal approach, the transperitoneal approach was commonly preferred. The choice of the clamping location depended on the anatomy of the patient, but infrarenal ACC was preferred whenever possible. Before ACC, a bolus of heparin at a dose of 50–100 IU/kg was administered, and the activated clotting time (ACT) was measured after 5 min. The target ACT range was 200–220 s, and ACT monitoring was conducted every 30 min. If the ACT exceeded 220 s, no additional heparin was administered; however, if the ACT dropped below 200 s, additional heparin was administered. If feasible, preservation of the internal artery was sought, and the reimplantation of inferior mesenteric artery was considered in patients at a high risk of colonic ischemia.

### 2.4. Perioperative Assessment Protocol

The standard preoperative assessment for patients undergoing AAA surgery at our hospital included patient interviews, physical examinations, basic blood tests, chest X-rays, electrocardiography, and transthoracic echocardiography. Additional cardiac examinations, such as stress echocardiography or coronary angiography, were conducted only for patients suspected of serious coronary artery disease based on standard examination. If coronary angiography identified a lesion requiring immediate revascularization, the revascularization was performed before surgery unless an urgent AAA repair was required. Postoperative troponin testing was performed in all patients immediately after AAA surgery for screening purposes, but additional troponin testing was performed only when necessary, such as in cases where myocardial injury was suspected.

### 2.5. Definition of MINS

In this study, MINS was defined as a high-sensitive TnT elevation above the 99th percentile of the upper reference limit (URL) (>0.014 ng/mL) caused by ischemic etiology within 30 days after surgery, as previously described [7,9,10]. Since 2010, our hospital has used the Elecsys^®^ Troponin T High Sensitive assay (Roche Diagnostics, Mannheim, Germany) to measure TnT levels. All patients included in this study had their TnT values measured using the high-sensitive TnT assay. Thus, we set 0.014 ng/mL as the threshold for MINS according to the 99th URL of the device. TnT elevation due to non-ischemic causes was not included in MINS, as in previous studies [9,10].

### 2.6. Study Endpoints

The primary endpoints were 30-day and 1-year mortalities after open repair of AAA. The secondary endpoint was the overall survival rate after AAA open repair. In addition, the risk factors for the occurrence of MINS were investigated.

### 2.7. Statistical Analyses

Statistical analyses were performed with IBM SPSS Statistics for Windows version 23 (IBM Corp., Armonk, NY, USA) and MedCalc version 22.014 (MedCalc Software Bvba, Ostend, Belgium). The Kolmogorov–Smirnov test was used in assessing the normality of continuous variables. Since all continuous variables in this study did not follow a normal distribution, continuous variables are presented as medians (interquartile ranges [IQR]). Categorical variables are presented as numbers (percentages). The Mann–Whitney test was performed for comparing continuous variables, and the chi-square or Fisher’s exact test was performed for comparing categorical variables. Logistic regression analysis was performed to investigate the predictors of 30-day mortality, 1-year mortality, and MINS occurrence. Multivariate logistic regression analysis included the variables which had *p*-values <0.05 in the univariate analysis. The predictability is presented by odds ratio (OR) and its 95% confidence interval (CI). The variance inflation factor (VIF) was evaluated to prevent multicollinearity when conducting the multivariate analyses. Kaplan–Meier survival curves were plotted to assess the overall survival rate among patients with and without MINS, and the log-rank test was conducted to estimate the statistical difference between the groups. A *p*-value < 0.05 was considered as statistically significant.

## 3. Results

**Figure 1:**
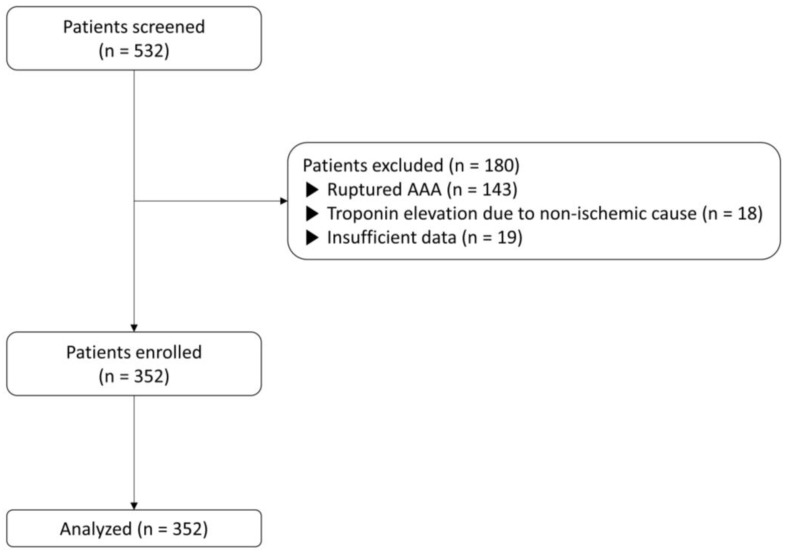
Flow diagram of the study.

The 532 patients who underwent AAA open repairs between September 2010 and April 2022 were screened for eligibility. We excluded 143 patients with ruptured AAA, 18 with troponin elevation due to non-ischemic causes (atrial fibrillation = 15, sepsis = 2, and acute decompensated heart failure = 1), and 19 with insufficient medical records. Finally, 352 patients were included in this study (Figure 1). All patients included in this study had troponin results within 3 days after surgery, whereas 16% (55/352) of patients had troponin results between 4 and 30 days after surgery. The median follow-up duration was 1402 [IQR 761, 2220] days.

In this study, MINS was diagnosed in 41.2% (145/352) of the patients, and 87% (126/145) of MINS were diagnosed within 2 days after surgery (Supplementary Figure S1). Table 1 summarizes the baseline and intraoperative characteristics among patients with and without MINS. Patients with MINS were significantly older (76 [69, 80] vs. 70 [63, 76] years, *p* < 0.001) and had a lower BMI (22.9 [21.1, 25.2] vs. 24.1 [21.9, 26.1] kg/m^2^, *p* = 0.026) than those without MINS. The prevalence of CKD (15.9% vs. 3.4%, *p* < 0.001), ESRD (3.4% vs. 0%, *p* = 0.011), and preoperative anemia (55.9% vs. 35.3%, *p* < 0.001) was higher among patients with MINS. The AAA diameter was significantly larger in patients with MINS (70 [56, 85] vs. 60 [52, 70] mm, *p* < 0.001). Preoperative hemoglobin levels (12.3 [10.6, 13.9] vs. 13.3 [12.3, 14.3] g/dL, *p* < 0.001), platelet counts (189,000 [151,500, 241,000] vs. 207,000 [170,000, 244,000]/μL, *p* = 0.014), C-reactive protein levels (4.6 [1.3, 22.5] vs. 1.8 [0.8, 4.8] mg/L, *p* < 0.001), and creatinine levels (1.01 [0.83, 1.38] vs. 0.89 [0.75, 1.05] mg/dL, *p* < 0.001) showed significant differences between the two groups.

Table 2 summarizes the postoperative morbidity and mortality among the patients with and without MINS. The lengths of hospital stay (11 [9, 17] vs. 9 [7, 13] days, *p* < 0.001) and ICU stay (1 [1, 3] vs. 1 [1, 1] days, *p* < 0.001) were significantly longer among patients with MINS. The incidences of AKI (35.2% vs. 19.3%, *p* < 0.001) and myocardial infarction (2.8% vs. 0%, *p* = 0.028) were significantly higher among patients with MINS. In addition, the incidences of 30-day mortality (5.5% vs. 0.5%, *p* = 0.004) and 1-year mortality (8.3% vs. 1.4%, *p* = 0.002) were significantly higher among patients with MINS.

Table 3 presents the results of the logistic regression analysis for 30-day mortality after AAA open repair. In the univariate analysis, BMI and MINS had significant OR for 30-day mortality. In the multivariate analysis, which included variables with significant OR in the univariate analysis, only MINS remained an independent predictor of 30-day mortality (OR: 10.440, 95% CI: 1.278–85.274, *p* = 0.029).

Table 4 shows the logistic regression analysis between the chosen variables and 1-year mortality after AAA open repair. BMI, history of cerebrovascular accident, preoperative anemia, and MINS showed significant OR (*p* < 0.05) for 1-year mortality in the univariate analysis. In the multivariate analysis, BMI (OR: 0.787, 95% CI: 0.636–0.974, *p* = 0.028), history of cerebrovascular accident (OR: 6.692, 95% CI: 2.037–21.986, *p* = 0.002), and MINS (OR: 5.189, 95% CI: 1.357–19.844, *p* = 0.016) remained independent predictors of 1-year mortality.

**Figure 2:**
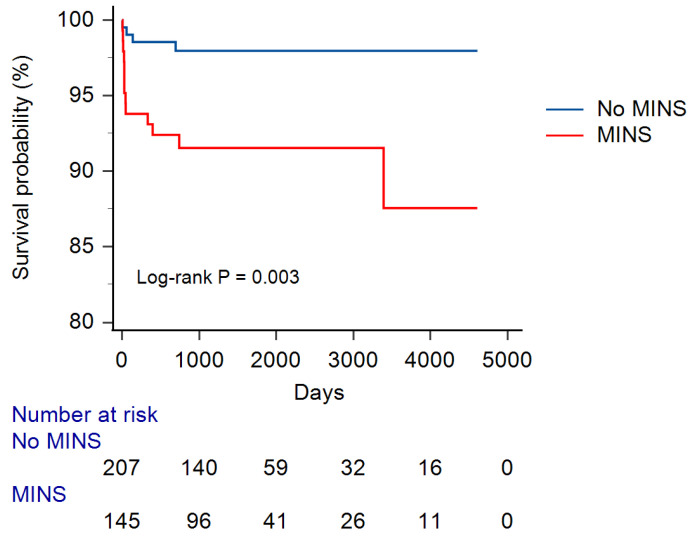
Kaplan–Meier survival curves according to the occurrence of MINS. The red line represents the survival curves of the patients with MINS, and the blue line represents the survival curves of those without MINS. MINS, myocardial injury after non-cardiac surgery.

Figure 2 presents the Kaplan–Meier survival curves for overall mortality. The log-rank test demonstrated that the overall survival rate was significantly lower among patients with MINS compared to those without MINS (*p* = 0.003).

Table 5 presents the logistic regression analysis for the occurrence of MINS. The univariate analysis showed that age, history of CKD, preoperative anemia, and AAA size have significant OR (*p* < 0.05) in predicting MINS. In the multivariate analysis, age (OR: 1.052, 95% CI: 1.023–1.081, *p* < 0.001), CKD (OR: 3.755, 95% CI: 1.471–9.581, *p* = 0.006), and AAA size (OR: 1.027, 95% CI: 1.013–1.042, *p* < 0.001) remained independent predictors of MINS.

## 4. Discussion

This study demonstrated that MINS is an independent risk factor of 30-day and 1-year mortalities in AAA open repair. Patients with MINS had lower survival rates and higher postoperative morbidity rates than those without MINS. Furthermore, age, history of CKD, and AAA size were identified as risk factors for the occurrence of MINS after AAA open repair.

MINS has been reported to be closely associated with mortality in patients undergoing vascular surgery [11]. However, the predictive power of MINS in AAA open repair remains underexplored. A few studies conducted before the introduction of the structured concept of MINS have investigated myocardial injury during AAA open repair [13,14]. However, in these studies, the current diagnostic criteria of MINS were not used, the number of enrolled patients was small, covariates were not considered, and long-term mortality was not analyzed. The current study identified MINS using the recently proposed diagnostic criteria [9,10] and revealed the independent prognostic role of MINS for long-term mortality, as well as short-term mortality, after AAA open repair.

In this study, the incidence of MINS (41%) exceeded those reported in studies on heterogeneous non-cardiac surgery. A previous study by Vascular Events in Non-Cardiac Surgery Patient Cohort Evaluation group reported an incidence of MINS of approximately 8% after major non-cardiac surgery [15]. Furthermore, a meta-analysis estimated an incidence of MINS of approximately 20% [16]. However, the reported incidence of myocardial injury in AAA open repair was 47% [13], which is consistent with our findings. The high incidence of MINS in AAA open repair lacks a clear explanation; however, this may be attributed to the pathogenesis and surgical characteristics of AAA. Traditionally, chronic inflammation and atherosclerosis have been described as the mechanisms underlying AAA development. Chronic inflammation disrupts the aortic wall and leads to aortic aneurysm formation [17,18]. However, the inflammatory response usually occurs systemically; therefore, patients with AAA may have been cumulatively more exposed to inflammatory mediators than other patients. Therefore, these patients may be more vulnerable to stress responses induced by surgical trauma and anesthesia, such as cortisol, catecholamines, and inflammatory cytokines, which could lead to MINS [10,19]. On the other hand, some researchers suggested that AAA is caused by excessive positive remodeling of vessels in response to atherosclerosis [17,20]. Consistent with this argument, atherosclerosis is an important risk factor for AAA [21], and a previous study reported high prevalence of CAD in AAA patients [22]. Thus, patients with AAA can be assumed to be more susceptible to myocardial injury due to a high prevalence of underlying CAD. In addition, the surgical characteristics of AAA open repair may also contribute to the development of MINS. Surgical procedures for AAA open repair include ACC, which causes ischemia–reperfusion injury by producing reactive oxygen species, inflammatory molecules, and catecholamines [23]. Oxygen radicals cause myocardial edema and dysfunction by increasing the permeability of vessels [24,25], and ischemia–reperfusion activates the coagulation system, leading to microthrombus formation [26]. Furthermore, AAA open repair is accompanied by severe hemodynamic fluctuations, especially when applying and removing the ACC [27]. Hemodynamic fluctuation is an important cause of MINS, causing a supply–demand mismatch in the myocardium [19].

The current study revealed not only higher mortality but also increased postoperative complications among patients with MINS, including AKI, cerebrovascular accident, and myocardial infarction. MINS is defined as a myocardial injury which occurs for up to 30 days, and postoperative morbidity may frequently occur within 30 days. Thus, the time order between MINS and morbidity may vary depending on the patient. Although 87% of patients experienced MINS within 2 days after surgery in this study, it is difficult to estimate the predictability between MINS and postoperative complications. Nevertheless, it is clear that MINS is associated with poor recovery after AAA open repair, and identifying MINS may help screen patients with poor recovery.

In the current study, age, history of CKD, and AAA diameter were independent risk factors for the occurrence of MINS. This result is consistent with previous studies that reported age and CKD as independent risk factors for MINS [10,15,16,28]. Interestingly, this study found that AAA diameter, which is a disease-specific risk stratification tool in AAA, was an independent risk factor of MINS. To the best of our knowledge, this is the first study to report a significant correlation between AAA size and MINS. Patients with larger AAA may have more severe atherosclerosis owing to longer disease progression [29], which could be the reason for the higher incidence of MINS among these patients.

MINS can result from various causes, including surgical trauma, anesthesia, hemodynamic instability, or endothelial dysfunction [10]. Surgical trauma can cause inflammatory reactions and stress responses, potentially leading to MINS [10]. However, it has not been clearly revealed until what time point the surgery is directly related to MINS. An animal study demonstrated that the tissue concentration of IL-1β and IL-6, induced by surgical trauma, peaked 1 to 2 days after surgery in a rat model [30]. Additionally, a study involving patients undergoing knee and hip arthroplasty revealed that the serum concentration of IL-6 followed a pattern of peaking 24–48 h after surgery and decreasing thereafter [31]. Considering the time course of inflammatory cytokines after surgery, the impact of surgery on MINS is expected to be most significant within first 2 days after surgery. However, this aspect has not been thoroughly investigated, and further research is warranted.

MINS is usually asymptomatic and can easily go undiagnosed. Previous studies reported that only 7%–18% of patients with MINS had symptoms, and only 25%–35% of MINS cases showed electrocardiogram changes [5,7,9,15]. Therefore, identifying MINS requires routine troponin testing in all patients regardless of ischemic signs or symptoms [10,32]. In particular, AAA repair is classified as a high-risk surgery according to the Revised Cardiac Risk Index [33], and myocardial infarction is a common cause of mortality in AAA open repair [34]. Considering the high incidence of MINS among patients undergoing AAA open repair, perioperative troponin tests may be a reasonable screening tool in AAA open repair.

International guidelines recommend troponin testing after non-cardiac surgery in high-risk patients. The Canadian Cardiovascular Society recommends daily troponin testing for 48–72 h after surgery in high-risk patients [32], and the European Society of Cardiology recommends troponin testing 24 and 48 h after surgery in high-risk patients [35]. These guidelines are based on the previous studies that showed most myocardial infarctions or MINS occur within 2 days after surgery [5,10,36]. In the current study, 87% (126/145) of MINS cases were diagnosed within 2 days after surgery, which is consistent with previous findings. Therefore, conducting troponin tests within 2–3 days after AAA surgery may be a reasonable strategy.

Several researchers have argued for enhanced risk assessment in AAA surgery, but the risk–benefit ratio must be carefully considered. Early studies highlighted the predictive value of dobutamine stress echocardiography before aortic surgery [37,38], and Sigl et al. recently reported improved 30-day outcomes after AAA surgery with extended cardiac assessment using dobutamine stress echocardiography [39]. However, stress echocardiography may cause side effects such as arrhythmia or hypotension [40,41], and some studies question its benefit in AAA surgery [42,43]. On the other hand, early studies reported dipyridamole–thallium scanning as a valuable predictor of outcomes after AAA surgery [44,45]. However, later studies indicated that dipyridamole–thallium scan was not superior to standard examinations [46,47], and many patients experienced side effects such as dizziness or chest tightness [48]. In contrast to stress testing, the troponin test can be performed with a simple blood test and has minimal associated risks. Moreover, a previous study reported the cost-effectiveness of troponin testing for screening MINS [49]. Therefore, conducting perioperative troponin tests in AAA surgery may serve as a cost-effective tool for screening high-risk patients.

In a previous study by Ali et al. [13], CAD prevalence was higher in patients experiencing myocardial injury after AAA surgery compared to those who did not. However, in our current study, there was no significant difference in CAD prevalence between patients with MINS and those without. This discrepancy may be attributed to variations in the cardiac risk assessment protocols employed in the two studies. In our study, preoperative stress testing was exclusively conducted on patients suspected of having serious coronary artery disease in standard examination. In contrast, Ali et al. [13] routinely performed stress testing in patients presenting cardiac symptoms. Consequently, some patients in our study may have had undiagnosed CAD, potentially affecting the accuracy of CAD prevalence, while Ali et al.’s study may have provided more accurate information about the prevalence of CAD. However, there exists controversy regarding the routine implementation of preoperative stress testing, necessitating careful consideration of the risk–benefit ratio.

Lower BMI was also an independent risk factor for 1-year mortality in the current study. Although obesity is a well-known risk factor for cardiovascular diseases [50,51,52], several recent studies have reported the “obesity paradox,” suggesting that obese patients have a better prognosis in various cardiovascular diseases, including chronic heart failure [53,54], CAD [55,56], and acute myocardial infarction [57]. In particular, previous studies on heart failure have reported that the relationship between mortality and BMI showed a U-shape [54]. A meta-analysis by Milajerdi et al. [58] reported that mortality among patients with heart failure increased when the BMI was <25 kg/m^2^ or >29 kg/m^2^. Another meta-analysis by Padwal et al. [59] reported that mortality increased when the BMI was <30 kg/m^2^ or >35 kg/m^2^ in heart failure patients. The patients included in the current study had a relatively low BMI of median 23.5 [IQR 21.5, 25.9] kg/m^2^, which corresponds to the anterior descending curve of the U shape; therefore, patients with higher BMI showed lower mortality in this study. To accurately estimate the relationship between BMI and mortality after AAA open repair, further studies that include patients with obesity are necessary.

This study has several limitations. First, the retrospective nature introduces the possibility of inaccurate data or missing values, potentially causing biases. However, the troponin test results, which are the most important variable in this study, were preserved as laboratory test records, ensuring the reliability of the dates and values. Moreover, the exclusion of 19 patients due to insufficient data in this study is a relatively small number. Secondly, the retrospective design hinders control over covariates, which could potentially act as confounding factors. However, this study employed multivariate regression analyses to minimize biases from covariates. Third, this study has limitations as a single-center study, including the lack of generalizability. While this study analyzed 352 AAA patients, a relatively large sample size for investigating AAA surgery, the competency of medical staff, perioperative protocols, or patient population could influence the results. To validate our findings, additional multicenter studies involving diverse populations are warranted. Fourth, the incidence of mortality was relatively small; therefore, the statistical power may be possibly weak when performing multivariate logistic regression analysis. Additional studies with larger populations are warranted to precisely estimate predictors of mortality. Conversely, the number of MINS cases was relatively large in this study, including 145 patients, potentially providing high statistical power for the analysis of predictors of MINS.

## 5. Conclusions

MINS is a common complication of AAA open repair and is an independent risk factor of 30-day and 1-year mortalities. Patients with MINS have lower survival rates than those without MINS. Conducting perioperative troponin tests to identify MINS in AAA open repair may be helpful in screening patients at risk of mortality. However, additional studies involving larger populations and diverse institutions are needed.

## Figures and Tables

**Table 1:** Baseline and intraoperative characteristics according to the presence of MINS.

	No MINS (*n* = 207)	MINS (*n* = 145)	*p*-Value
Sex (female)	41 (19.8%)	29 (20.0%)	0.964
Age (years)	70 (63, 76)	76 (69, 80)	<0.001
BMI (kg/m^2^)	24.1 (21.9, 26.1)	22.9 (21.1, 25.2)	0.026
Smoking	55 (26.6%)	31 (21.4%)	0.265
Hypertension	144 (69.6%)	111 (76.6%)	0.149
Diabetes mellitus	32 (15.5%)	22 (15.2%)	0.941
Cerebrovascular accident	22 (10.6%)	21 (14.5%)	0.277
Coronary artery disease	45 (21.7%)	36 (24.8%)	0.498
Previous PCI	35 (16.9%)	25 (17.2%)	0.935
PAOD	7 (3.4%)	5 (3.4%)	>0.999
COPD	6 (2.9%)	3 (2.1%)	0.742
Chronic kidney disease	7 (3.4%)	23 (15.9%)	<0.001
ESRD	0	5 (3.4%)	0.011
Anemia	73 (35.3%)	81 (55.9%)	<0.001
AAA size (mm)	60 (52, 70)	70 (56, 85)	<0.001
Preoperative medications			
Beta-blockers	64 (30.9%)	49 (33.8%)	0.570
Calcium channel blockers	80 (38.6%)	59 (40.7%)	0.700
RAS inhibitors	84 (40.6%)	61 (42.1%)	0.780
Statins	95 (45.9%)	66 (45.5%)	0.944
Diuretics	22 (10.6%)	30 (20.7%)	0.009
Preoperative laboratory results			
WBC count (/μL)	6860 (5820, 8690)	7580 (6115, 9660)	0.005
Hemoglobin (g/dL)	13.3 (12.3, 14.3)	12.3 (10.6, 13.9)	<0.001
Platelet count (/μL)	207,000 (170,000, 244,000)	189,000 (151,500, 241,000)	0.014
C-reactive protein (mg/L)	1.8 (0.8, 4.8)	4.6 (1.3, 22.5)	<0.001
BUN (mg/dL)	16.4 (13.1, 19.9)	20.2 (16.0, 25.5)	<0.001
Creatinine (mg/dL)	0.89 (0.75, 1.05)	1.01 (0.83, 1.38)	<0.001
eGFR (mL/min.1.73 m^2^)	85 (69, 93)	70 (48, 85)	<0.001
Intraoperative data			
Administered fluid (mL)	2550 (1900, 3400)	2500 (1900, 3300)	0.902
Urine output (mL)	305 (150, 500)	275 (135, 489)	0.317
Cell saver (mL)	226 (132, 423)	238 (112, 384)	0.737
Bleeding (mL)	500 (500, 825)	500 (500, 925)	0.604
Operative time (min)	132 (104, 166)	136 (105, 173)	0.659
Anesthesia time (min)	205 (175, 235)	195 (170, 235)	0.413
ACC time (min)	40 (30, 53)	40 (30, 50)	0.257

Values are presented as the median (interquartile range) or number of patients (%). MINS, myocardial injury after non-cardiac surgery; BMI, body mass index; PCI, percutaneous coronary intervention; PAOD, peripheral arterial occlusive disease; COPD; chronic obstructive pulmonary disease; ESRD, end-stage renal disease; AAA, abdominal aortic aneurysm; RAS, renin–angiotensin system; WBC, white blood cells; BUN, blood urea nitrogen; eGFR, estimated glomerular filtration rate; ACC, aortic cross-clamp.

**Table 2:** Postoperative morbidity and mortality according to the presence of MINS.

	No MINS (*n* = 207)	MINS (*n* = 145)	*p*-Value
Hospital stay (day)	9 (7, 13)	11 (9, 17)	<0.001
ICU stay (day)	1 (1, 1)	1 (1, 3)	<0.001
MV duration (h)	0 (0, 0)	0 (0, 4)	<0.001
Cerebrovascular accident	2 (1.0%)	1 (0.7%)	>0.999
Acute kidney injury	40 (19.3%)	51 (35.2%)	0.001
Myocardial infarction	0	4 (2.8%)	0.028
30-day mortality	1 (0.5%)	8 (5.5%)	0.004
1-year mortality	3 (1.4%)	12 (8.3%)	0.002

Values are presented as the median (interquartile range) or number of patients (%). MINS, myocardial injury after non-cardiac surgery; ICU, intensive care unit; MV, mechanical ventilation.

**Table 3:** Logistic regression analysis for 30-day mortality after open repair of abdominal aortic aneurysm.

	Univariate		Multivariate	
Variables	OR (95% CI)	*p*-Value	OR (95% CI)	*p*-Value
Age	1.039 (0.964–1.120)	0.317		
BMI (kg/m^2^)	0.771 (0.610–0.974)	0.029	0.801 (0.632–1.015)	0.066
Hypertension	0.293 (0.077–1.116)	0.072		
Diabetes mellitus	0.684 (0.084–5.582)	0.723		
Cerebrovascular accident	2.105 (0.423–10.476)	0.364		
Coronary artery disease	1.699 (0.415–6.949)	0.461		
Chronic kidney disease	0.000 (-)	0.998		
Anemia	4.667 (0.956–22.792)	0.057		
AAA size (mm)	1.030 (0.998–1.063)	0.063		
MINS	12.029 (1.488–97.260)	0.020	10.440 (1.278–85.274)	0.029

Values are presented as odds ratio (95% confidential interval). OR, odds ratio; CI, confidence interval; BMI, body mass index; AAA, abdominal aortic aneurysm; MINS, myocardial injury after non-cardiac surgery.

**Table 4:** Logistic regression analysis for 1-year mortality after open repair of abdominal aortic aneurysm.

	Univariate		Multivariate	
Variables	OR (95% CI)	*p*-Value	OR (95% CI)	*p*-Value
Age	1.037 (0.978–1.100)	0.220		
BMI (kg/m^2^)	0.781 (0.650–0.938)	0.008	0.787 (0.636–0.974)	0.028
Hypertension	0.751 (0.250–2.256)	0.610		
Diabetes mellitus	0.843 (0.185–3.847)	0.826		
Cerebrovascular accident	5.405 (1.821–16.045)	0.002	6.692 (2.037–21.986)	0.002
Coronary artery disease	1.228 (0.380–3.965)	0.731		
Chronic kidney disease	2.870 (0.763–10.797)	0.119		
Anemia	3.731 (1.164–11.956)	0.027	2.041 (0.584–7.136)	0.264
AAA size (mm)	1.011 (0.982–1.040)	0.454		
MINS	6.135 (1.699–22.152)	0.006	5.189 (1.357–19.844)	0.016

Values are presented as odds ratio (95% confidential interval). OR, odds ratio; CI, confidence interval; BMI, body mass index; AAA, abdominal aortic aneurysm; MINS, myocardial injury after non-cardiac surgery.

**Table 5:** Logistic regression analysis for MINS occurrence after open repair of abdominal aortic aneurysm.

	Univariate		Multivariate	
Variables	OR (95% CI)	*p*-Value	OR (95% CI)	*p*-Value
Age	1.061 (1.035–1.089)	<0.001	1.052 (1.023–1.081)	<0.001
BMI (kg/m^2^)	0.940 (0.879–1.005)	0.068		
Hypertension	1.428 (0.879–2.320)	0.150		
Diabetes mellitus	0.978 (0.542–1.764)	0.941		
Cerebrovascular accident	1.424 (0.751–2.700)	0.279		
Coronary artery disease	1.189 (0.720–1.962)	0.498		
Chronic kidney disease	5.386 (2.244–12.928)	<0.001	3.755 (1.471–9.581)	0.006
Anemia	2.323 (1.505–3.587)	<0.001	1.413 (0.852–2.345)	0.180
AAA size (mm)	1.031 (1.017–1.045)	<0.001	1.027 (1.013–1.042)	<0.001

Values are presented as odds ratio (95% confidential interval). OR, odds ratio; CI, confidence interval; BMI, body mass index; AAA, abdominal aortic aneurysm; MINS, myocardial injury after non-cardiac surgery.

## Data Availability

The data presented in this study are available on request from the corresponding author.

## Supplementary Materials

The following supporting information can be downloaded at: https://www.mdpi.com/article/10.3390/jcm13040959/s1, Supplementary Figure S1: The timing of diagnosis of myocardial injury after non-cardiac surgery (MINS).
