# Supplementary material for: Myocardial Injury after Non-Cardiac Surgery in Patients Who Underwent Open Repair for Abdominal Aortic Aneurysm: A Retrospective Study
Source: J Clin Med. 2024 Feb 7;13(4):959. doi: 10.3390/jcm13040959 (PMC10888606; doi:10.3390/jcm13040959)
Supplement: Supplementary file 1 [file jcm-13-00959-s001.zip › jcm-2845050-supplementary.pdf]

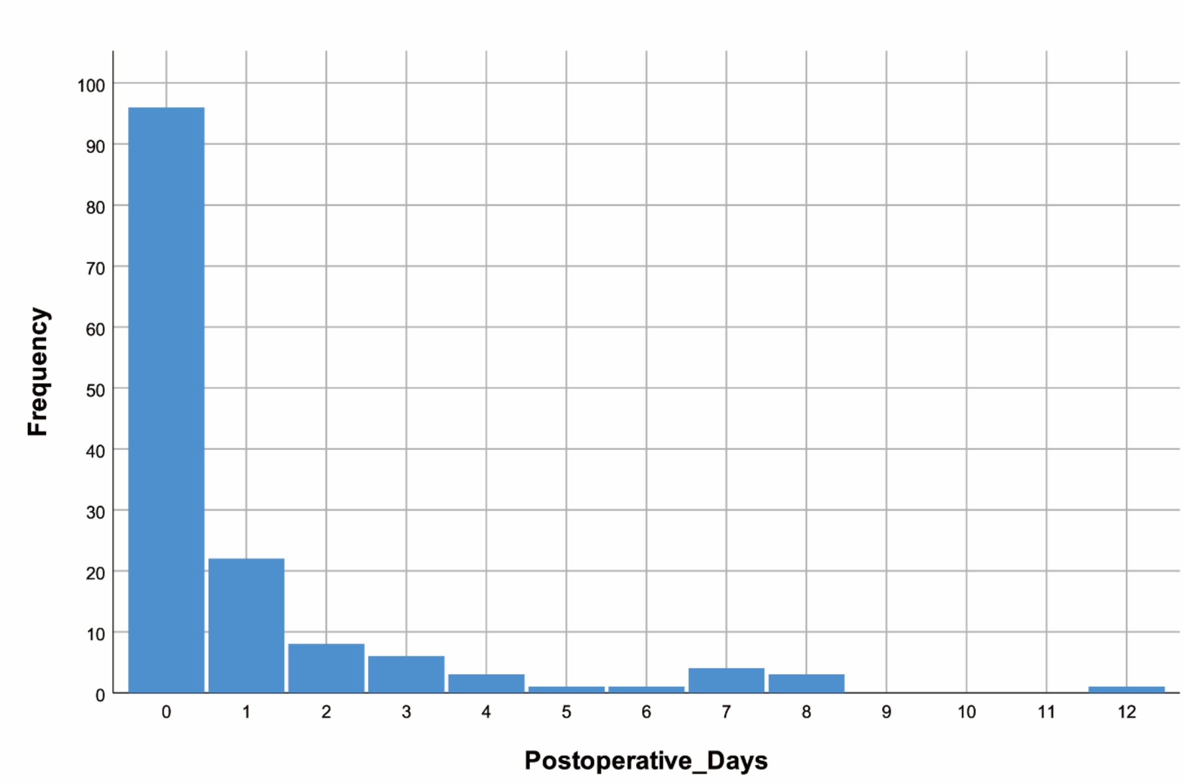

Supplementary Figure S1. The timing of diagnosis of myocardial injury after non-cardiac surgery (MINS). Frequency indicates the number of patients diagnosed with MINS. No patient was diagnosed with MINS on days 13–30.
